# Estimated Fluoride Doses from Toothpastes Should be Based on Total Soluble Fluoride

**DOI:** 10.3390/ijerph10115726

**Published:** 2013-11-01

**Authors:** Maria José L. Oliveira, Carolina C. Martins, Saul M. Paiva, Livia M. A. Tenuta, Jaime A. Cury

**Affiliations:** 1 Department of Paediatric Dentistry and Orthodontics, School of Dentistry, Universidade Estadual de Montes Claros (UNIMONTES), Campus Darcy Ribeiro, Vila Mauricéia, Montes Claros, MG 39410-089, Brazil; E-Mail: lagesdeoliveira@gmail.com; 2 Department of Paediatric Dentistry, School of Dentistry, Universidade Federal de Minas Gerais (UFMG), Avenida Antônio Carlos 6627, Belo Horizonte, MG 31270-901, Brazil; E-Mail: smpaiva@uol.com.br; 3 Department of Physiological Sciences, Piracicaba Dental School, University of Campinas (UNICAMP), Avenida Limeira 901, Piracicaba, SP 13414-903, Brazil; E-Mails: litenuta@fop.unicamp.br (L.M.A.T.); jcury@fop.unicamp.br (J.A.C.)

**Keywords:** fluorides, dentifrices, toothpastes, dental fluorosis, fluorosis risk

## Abstract

The fluoride dose ingested by young children may be overestimated if based on levels of total fluoride (TF) rather than levels of bioavailable fluoride (total soluble fluoride—TSF) in toothpaste. The aim of the present study was to compare doses of fluoride intake based on TF and TSF. Fluoride intake in 158 Brazilian children aged three and four years was determined after tooth brushing with their usual toothpaste (either family toothpaste (*n =* 80) or children’s toothpaste (*n =* 78)). The estimated dose (mg F/day/Kg of body weight) of TF or TSF ingested was calculated from the chemical analysis of the toothpastes. Although the ingested dose of TF from the family toothpastes was higher than that from the children’s toothpastes (0.074 ± 0.007 and 0.039 ± 0.003 mg F/day/Kg, respectively; *p <* 0.05), no difference between types of toothpaste was found regarding the ingested dose based on TSF (0.039 ± 0.005 and 0.039 ± 0.005 mg F/day/Kg, respectively; *p >* 0.05). The fluoride dose ingested by children from toothpastes may be overestimated if based on the TF of the product. This finding suggests that the ingested dose should be calculated based on TSF. Dose of TSF ingested by children is similar whether family or children’s toothpaste is used.

## 1. Introduction

The regular use of fluoridated toothpaste has been associated with a decline in dental caries in both developed and developing countries [1,2]. While the benefits of the use of such toothpaste by children and adolescents are well established [3], fluoridated toothpaste is also considered a risk factor for dental fluorosis [4].

Dental fluorosis is caused by the effect of fluoride ingested during the formation of tooth enamel. The severity of this condition depends on the dose (mg F/day/Kg of body weight) to which the child has been subjected [5]. The literature considers a dose of 0.05 to 0.07 mg F/day/Kg to be an acceptable range in terms of adequate caries control and the avoidance of unsightly (moderate) dental fluorosis [6]. Although this value has been extensively used as a reference to estimate the risk of fluorosis from toothpaste or the relative contribution of fluoride intake from toothpaste compared with fluoride intake from drinking water, longitudinal studies have not found a strong association between this dose and the development of dental fluorosis [7,8]. Furthermore, fluorosis is not resultant from the dose of fluoride ingested, but from the fraction absorbed in the gastrointestinal tract (bioavailability).

The amount of bioavailable fluoride in toothpaste depends on the type of fluoride salt and abrasive used in its composition [9,10,11,12]. Due to the incompatibility of NaF, SnF_2_ and even amine fluorides with calcium-based abrasives, silica (SiO_2_) particles have been employed. In such formulations, all fluoride is chemically soluble [13,14,15] so as to be effective against the development of caries [16]. However, if ingested during tooth brushing, all fluoride becomes bioavailable and has a systemic effect, which increases the risk of fluorosis [17].

Toothpastes containing calcium-based abrasives, such as calcium carbonate (CaCO_3_) or dehydrated calcium phosphate (CaH_2_PO_4_·2H_2_O), are formulated using sodium monofluorophosphate (MFP). Although the MFP ion is more compatible than the fluoride ion with abrasives containing Ca, part of the total fluoride in these formulations is insoluble [14,15] and therefore only partially bioavailable [9,10,11]. When the level of fluoride intake from toothpaste is estimated in children, it is important to take into account the amount of bioavailable fluoride in the formulation. The dose may be overestimated when the total fluoride (TF) declared on the label is considered and the toothpaste contains MFP/Ca-salts. This is extremely relevant in developing countries, where toothpastes containing MFP/Ca-salts are usually formulated with a higher fluoride concentration than those containing NaF/SiO_2_ [14,18,19,20]. If the total soluble fluoride (TSF) in the formulation is used to calculate fluoride intake, both types of toothpaste may be equivalent in terms of fluorosis risk.

In some developing countries, MFP/CaCO_3_ formulations with TF of 1,400 to 1,500 µg F/g are often used by children in families with a lower socioeconomic status, while NaF/SiO_2_-based toothpastes, which usually contain 1,000 to 1,100 µg F/g, are used by children of families with a higher socioeconomic status [21]. While some studies show that family toothpastes (*i.e.*, those used both by children and the rest of the family) can contribute to a significantly higher F intake than children’s toothpastes (those used exclusively by the children of a family) [22,23,24], other studies have found divergent results [21,25,26]. However, the majority of the papers cited do not specify the type of fluoride considered for the estimation of F intake from toothpastes [22,23,24,26] and only two studies considered F intake based on TSF determined in the chemical analysis of the toothpastes [21,25].

Therefore, the hypothesis of the present study is that the reported dose of fluoride that children ingest from toothpaste is overestimated if the TF of the formulation is considered rather than TSF.

## 2. Experimental Section

### 2.1. Ethical Considerations and Sampling

This study (NCT01568541) received approval from the Human Research Ethics Committee of the Federal University of Minas Gerais, Brazil (protocol #278/07). All parents/guardians received information regarding the objectives of the study and signed terms of informed consent. The toothpastes taken for analysis were replaced with new toothpastes.

Two hundred eight children aged nine to 48 months attending four private and four public kindergartens in Montes Claros, MG, Brazil, were enrolled in the present study. Forty-nine children were excluded: nine because they used non-fluoridated toothpastes, four because they did not complete the entire data collection process and 36 because they were aged nine to 35 months. Thus, the final sample comprised 159 children aged 36 to 48 months (weight = 18.8 ± 6.2 Kg (mean ± standard deviation) and age = 43.4 ± 4.3 months (mean ± standard deviation)).

### 2.2. Experimental Design

**Figure 1:**
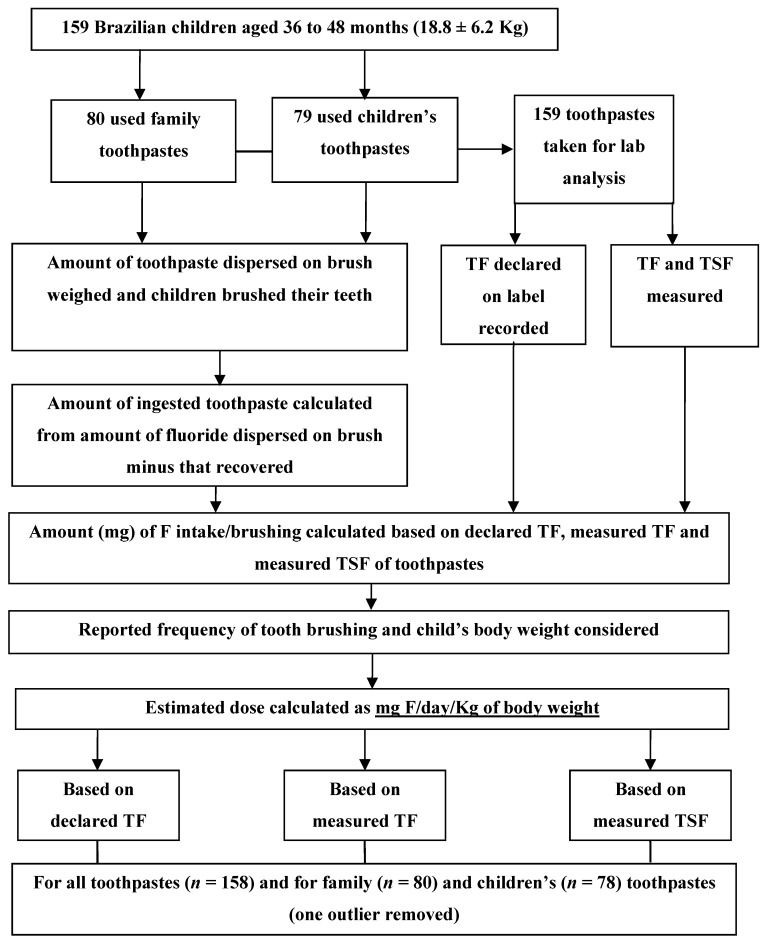
Flow chart of study design.

Experimental protocol followed design proposed by Guha-Chowdhury *et al*. [27] The parents/guardians attended a meeting at the kindergarten and brought the toothbrush and toothpaste that the child used at home. The children brushed their teeth as they usually did at home. The amount of toothpaste used was weighed and the amounts of fluoride ingested as total fluoride (TF) and total soluble fluoride (TSF) were calculated based on an analysis of the toothpaste and the TF declared on the label (Figure 1). The toothpastes were classified [21] as: family (those used by everyone in the child’s family, mostly MFP/calcium carbonate containing 1,500 ppm F) or children’s (those used exclusively by the child of the family, mostly NaF/silica based containing 1,100 ppm F). The amount of fluoride ingested was multiplied by the frequency of tooth brushing reported by the parents and divided by the child’s body weight. The fluoride dose (mg F/day/Kg of body weight) was determined using the concentration of TF declared on the label as well as the TF and TSF found in the analysis of the toothpastes. The doses based on the declared TF, measured TF and TSF were analysed statistically for all toothpastes. Differences among doses based on declared TF, measured TF and TSF were statistically compared for each type of toothpaste (family (*n =* 80) and children’s (*n =* 79)) and between the two (Figure 1).

### 2.3. Toothpastes Used

Eighty children used family toothpastes and 79 used children’s toothpastes (Figure 1). While most family toothpastes contained MFP/CaCO_3_, children’s toothpastes contained NaF/silica. Fluoride concentrations were chemically determined [14] and are displayed in Table 1. The TF concentration in the toothpastes was in agreement with that declared on the label for all toothpastes. However, the TSF concentration found in the analysis of the family toothpastes was lower than the measured or declared TF. TF represents the sum of soluble fluorides (fluoride ion supplied by the NaF and MFP ion) and insoluble fluoride bound to the abrasive (usually found in MFP/CaCO_3_ toothpaste). TSF is the sum of soluble fluorides (fluoride ion and MFP ion).

**Table 1:** Concentration (ppm F) of total fluoride (TF) declared on label, TF found in lab analysis and total soluble fluoride (TSF) measured in toothpastes according to type of toothpaste (mean ± standard deviation; *n*).

Toothpaste	F concentration (ppm F)		
	Declared TF	Measured TF	Measured TSF
Family * (*n* = 80)	1,424.3 ± 12.0	1,434.0 ± 16.2	971.6 ± 23.4
Children’s ** (*n* = 79)	1,074.9 ± 13.1	1,062.3 ± 12.4	1,070.7 ± 24.5
All (*n* = 159)	1,251.8 ± 14.6	1,250.5 ± 18.0	1,020.0 ± 17.3

* Used by whole family; ** used only by children.

### 2.4. Determination of Fluoride Intake

Each parent/child pair was taken to the WC of the kindergarten and the child was instructed to brush his/her teeth as he/she usually did at home. The amount of toothpaste dispersed on the toothbrush was weighed (±0.01 g). The child performed tooth brushing in his/her normal way with or without the aid of his/her parent. No formal instructions were given regarding tooth brushing technique. If the child requested to rinse his/her mouth, the researcher provided purified water for rinsing and a plastic cup to collect the expectorated residue. When the child rinsed his/her mouth and/or spat, the residues were collected in the plastic cup. The toothbrush was vigorously washed with purified water and the residue was collected in the same plastic cup containing the expectorated salivary residue. The mixture, denominated “brushing residue”, was homogenised. The volume was measured and an aliquot of 15 mL of the sample was frozen and stored for the subsequent determination of the fluoride concentration.

Duplicates of 0.25 mL of this mixture were transferred to assay tubes to which 0.25 mL of 2 M HCL were added. The tube was maintained at 45 °C for 1 h to hydrolyse the MFP and dissolve any insoluble fluoride bound to the abrasive elements of the toothpaste, if present in the brushing residue. The acid extract was neutralised with 0.5 mL of 1 M NaOH and buffered with 1.0 mL of TISAB [14]. The TF concentration in the sample was determined with an ion-selective electrode and the amount of non-ingested fluoride (mg) was calculated. As the TF in the toothpaste was known, the amount of non-ingested toothpaste was determined from the amount of non-ingested fluoride. The amount of ingested toothpaste was determined by subtracting the amount of non-ingested toothpaste from the amount placed on the toothbrush. Fluoride intake per brushing was determined from the amount of toothpaste ingested and the declared TF, measured TF and TSF. The dose (mg F/day/Kg of body weight) ingested was estimated from the frequency of tooth brushing reported and the weight of the child. All calculations were made using the EXCEL programme (Microsoft, Redmond, WA, USA).

### 2.5. Fluoride Analysis

The analysis of the toothpastes and brushing residues was performed using an ion-specific electrode (Orion 96-09) and an ion analyser (Thermo Scientific Orion, Chelmsford, MA USA), previously calibrated using F standards (final concentration of 0.0625, 0.125, 0.25, 0.5, 1.0, 2.0 and 4.0 ppm) prepared as samples [14]. For each analysis, a linear regression between the F concentration of the standards (µF/mL) and mV was created (r^2^ > 0.999) using the EXCEL programme (Microsoft) and used to calculate the F concentration in the samples. Mean coefficients of variation in duplicate analyses were less than 1%.

### 2.6. Statistical Analysis

Dose data was tested for normality using the Kolmogorov-Smirnov test and Levene’s test for variance homogeneity, which demonstrated non-normal distribution and non-homogeneous variance, respectively. The non-parametric Mann-Whitney test (α = 5%) was used to compare means of F intake between the children’s and family toothpastes as well as between declared TF, measured TF and TSF for each type of toothpaste separately and for all toothpastes. The *Statistical Package for Social Sciences* (SPSS for Windows, version 18.0, SPSS Inc., Chicago, IL, USA) was used for the analysis. One child who used a children’s toothpaste was detected as an outlier (dose = 0.7850 mg F/day/Kg of body weight) and was excluded from the sample (*n* = 158 children).

## 3. Results

The mean (±standard deviation) weight of the toothpaste used during brushing was 0.55 ± 0.36 g for children’s toothpaste and 0.59 ± 0.36 g for family toothpaste; this difference was non-significant (*p >* 0.05). No statistically significant difference was found in the mean age of the children who used family toothpaste (43.9 ± 4.5 months) (mean ± standard deviation) and those who used children’s toothpaste (42.8 ± 4.0 months) (*p* = 0.102, Mann-Whitney test). No statistically significant difference was found in the mean weight of the children who used family toothpaste (17.9 ± 5.2 Kg) and those who used children’s toothpaste (19.6 ± 7.0 Kg) (*p* = 0.111, Mann-Whitney test). Thus, both groups were homogeneous with regard to age and weight.

Table 1 displays the declared TF, measured TF and measured TSF of the toothpastes (ppm F). Family toothpastes had lower TSF (971.6 ± 23.4 ppm F) than declared TF (1,424.3 ± 12.0 ppm F) and measured TF (1,434.0 ± 16.2 ppm F). Children’s toothpastes had similar concentrations of F for declared TF, measured TF and measured TFS (1,074.9, 1,062.3 and 1,070.7 ppm F, respectively).

**Table 2:** Estimated dose of fluoride (mg F/day/Kg of body weight) based on total fluoride (TF) declared on label and TF and total soluble fluoride (TSF) measured in analyses according to type of toothpastes (mean ± standard deviation; *n*).

Toothpaste	Dose (mg F/day/Kg bw)		
	Based on declared TF	Based on measured TF	Based on measured TSF
Family * (*n =* 80)	0.074 ± 0.007 A,a	0.074 ± 0.007 A,a	0.039 ± 0.005 A,b
Children’s ** (*n =* 78)	0.040 ± 0.007 B,a	0.039 ± 0.003 B,a	0.039 ± 0.005 A,a
All (*n =* 158)	0.057 ± 0.004 a	0.057 ± 0.004a	0.039 ± 0.003 b

* Used by whole family; ** used exclusively by children; Mann-Whitney test for 2 independent samples; Means followed by distinct capital letters differ statistically (*p <* 0.05) between types of toothpastes (columns) and means followed by distinct lowercase letters differ statistically (*p <* 0.05) for each type of toothpaste (lines).

No statistically significant differences were found in the estimates of ingested fluoride when calculated using measured TF or declared TF (*p >* 0.05) for all toothpastes or in the separate analyses of family and children’s toothpastes (Table 2). However, the dose in the family toothpastes based on TSF (0.039 ± 0.005 mg F/day/Kg of body weight) was lower than that based on declared TF (0.074 ± 0.007 mg F/day/Kg of body weight) or measured TF (0.074 ± 0.007 mg F/day/Kg of body weight) (*p <* 0.05). Moreover, the dose ingested from the family toothpastes was higher than that from children’s toothpastes when based on declared or measured TF (*p <* 0.001), but the difference was non-significant when based on TSF (*p* = 0.255).

## 4. Discussion

Following the increase in the prevalence of dental fluorosis reported in the USA in the 1980s [28], a number of studies were conducted to evaluate sources of fluoride that contributed to fluoride intake among children at the age of fluorosis risk in addition to the known systemic effect of fluoride in drinking water. However, fluoride intake from toothpaste should consider that the degree of fluoride absorption in the gastrointestinal tract from ingested toothpaste depends on the timing of tooth brushing in relation to mealtimes [29,30], the composition of meals [31] and the formulation of the toothpaste [10,11]. Therefore, the failure to consider how much fluoride is absorbed in the gastrointestinal tract may lead to an overestimation of the dose that constitutes fluorosis risk. Indeed, no association has been found between the dose of ingested fluoride and fluorosis [7,8].

The World Health Organization considers MFP/CaCO_3_ formulations to be affordable toothpastes for caries control due to their low production cost [32]. The most popular toothpastes used by the children enrolled in this study were the children’s toothpaste Tandy^®^ and the family toothpaste Sorriso^®^ and Colgate MPA^®^ (Colgate-Palmolive Industrial Ltda, São Paulo, Brazil) [14].

Besides that, the amount of chemically soluble fluoride in these formulations should be considered when evaluating the risk of fluorosis. The present data show that the dose to which children are subjected can be overestimated when total soluble fluoride (TSF) is not considered in the calculation of F intake (Table 2). Moreover, previous data demonstrate a lower degree of fluoride bioavailability in calcium-based toothpastes [9,10,11,12], which are the top selling toothpastes [20].

Since the majority of children’s toothpastes are formulated with silica/NaF, similar concentrations of declared TF, measured TF and TSF were found. In the family toothpastes, mean measured TSF was lower than declared TF and measured TF (Table 1).

As demonstrated in Table 2, if TF declared on the label or measured TF are used to estimate F intake in children, as previous studies have done [22,23,24,26,33,34], family toothpastes can lead to significantly greater F intake than children’s toothpastes (0.074 and 0.039 mg F/day/Kg of body weight, respectively). This alone reaches the upper limit of the dose considered to constitute fluorosis risk (0.070 mg F/day/Kg of body weight) [6]. However, TF is the sum of TSF and insoluble fluoride in the toothpaste formulation [13,14,15,35]. When considering measured levels of TSF, no difference in F intake was found between the children’s and family toothpastes. Therefore, children using a MFP/carbonate toothpaste containing around 1,500 ppm of TF or a NaF/silica toothpaste containing around 1,100 ppm F would be subjected to a “safe” dose if one only considers fluoride intake from a toothpaste, as shown in a previous study [12]. Although the present study did not evaluate gastrointestinal absorption, a previous study showed that fluoride salivary excretion is proportionally correlated to fluoride ingestion of TSF but not TF for both formulations [12]. Urinary fluoride excretion is a good indicator for fluoride intake [36], and when aged and fresh toothpastes of these formulations were ingested, one year aged toothpaste MFP/carbonate containing 1,500 ppm of TF had lower urinary fluoride excretion when compared to NaF/silica toothpaste containing around 1,100 ppm F [12], perhaps due the lower TFS of the formulation after one year of aging [12]. It is important to mention that regardless of the type of toothpaste, toothpastes should contain at least 1,100 ppm F to prevent caries [3], and it is important for paediatric dentists and manufacturers to inform parents regarding the need to use small amounts of toothpaste [21] to minimize F intake.

The present study has three major implications—one methodological, one pragmatic in terms of home toothpaste use and one social in terms of public health and clinical practice: (1) TSF should be considered the methodological measure of F intake from toothpaste in children, as intake may be overestimated when TF is used; (2) It is impractical for each member of a family to use a different specific fluoride toothpaste due to the risk of fluorosis and the whole family can benefit from the use of a family toothpaste with MFP/CaCO_3_ even if it contains 1,500 ppm F, balancing the benefits and risks; (3) MFP/CaCO_3_-based toothpastes are considered by the World Health Organization to be affordable toothpastes due to their low production costs [32]. These toothpastes are often used by children in families with a lower socioeconomic status, whereas NaF/SiO_2_-based toothpastes usually contain 1,000 to 1,100 μg F/g and are the choice in families with a higher socioeconomic status in both developing [21] and developed countries [37].

## 5. Conclusions

Total Soluble Fluoride (TSF) should be considered for the measure of F intake from toothpaste in children, as intake may be overestimated when Total Fluoride (TF) is considered. Both family toothpastes with 1,500 ppm MFP/CaCO_3_ and children’s toothpastes with 1,100 ppm NaF/silica lead to a similar dose of ingested fluoride (TSF) in young children.
